# Plasma proteomic profiling and molecular clustering reveal immune‐defined prognostic subtypes in lung adenocarcinoma

**DOI:** 10.1002/ijc.70231

**Published:** 2025-11-07

**Authors:** Ujjwal Neogi, Anoop T. Ambikan, Kati Turkowski, Marc A. Schneider, Vanessa M. Beutgen, Johannes Graumann, Hauke Winter, Marek Bartkuhn, Werner Seeger, Soni S. Pullamsetti, Rajkumar Savai

**Affiliations:** ^1^ The Systems Virology Lab, Division of Clinical Microbiology, Department of Laboratory Medicine Karolinska Institutet Stockholm Sweden; ^2^ Institute for Lung Health (ILH) Justus Liebig University Giessen Giessen Germany; ^3^ Max Planck Institute for Heart and Lung Research, Member of the German Center for Lung Research (DZL) Member of the Cardio‐Pulmonary Institute (CPI) Bad Nauheim Germany; ^4^ Translational Lung Research Center Heidelberg (TLRC), Member of the German Center for Lung Research (DZL) Thoraxklinik at Heidelberg University Hospital Heidelberg Germany; ^5^ Translational Research Unit Thoraxklinik at Heidelberg University Hospital Heidelberg Germany; ^6^ Institute of Translational Proteomics & Core Facility Translational Proteomics, Biochemical/Pharmacological Centre University of Marburg Marburg Germany; ^7^ Department of Thoracic Surgery Thoraxklinik at Heidelberg University Hospital Heidelberg Germany; ^8^ Pulmonary Hypertension and Vascular Biology Research Group of Quebec Heart and Lung Institute, Department of Medicine Laval University Quebec Canada

**Keywords:** adenocarcinoma, high‐throughput proteomics, inflammatory response, precision medicine

## Abstract

Lung adenocarcinoma (LUAD) is a biologically and clinically heterogeneous disease that poses a major challenge for prognosis and treatment. In this study, we performed proteomic profiling in a cohort of 88 LUAD patients to identify molecular subgroups and investigate their clinical relevance. Unsupervised clustering of the proteomic data allowed us to identify two distinct patient groups with different demographic, clinical, and molecular characteristics. Cluster 1 consisted predominantly of older patients and showed increased expression of immune and inflammatory pathways, including significant enrichment of Tumor Necrosis Factor (TNF) and Toll‐like receptor signaling. This suggests a stronger innate immune response that may be associated with better disease control. In contrast, Cluster 2 was characterized by younger demographics, a higher proportion of female patients, and a greater frequency of smoking. This cluster showed reduced activation of immune‐related pathways and a significantly shorter time to disease recurrence, suggesting a more aggressive clinical course and poorer prognosis. The differential expression of immune pathways between clusters underscores the role of the tumor microenvironment in disease progression and response to treatment. Our results demonstrate the value of integrating proteomic and clinical data to identify biologically distinct LUAD subtypes. This molecular stratification can improve the understanding of tumor behavior and inform personalized treatment strategies. Thus, proteomic profiling is a promising tool to guide biomarker‐directed treatment of LUAD.

## Graphical abstract

What's new?

Lung adenocarcinoma shows considerable heterogeneity in disease progression and therapeutic response. Here, the authors analyzed tumor samples through proteomic profiling and applied network‐based clustering. They identified two distinct patient groups with different demographic, clinical, and molecular characteristics. One cluster of predominantly older patients showed increased expression of immune and inflammatory pathways, suggesting a stronger innate immune response. The other cluster of mainly younger, female smokers showed reduced immune signaling and a more aggressive phenotype. The integration of blood‐based proteomic data with clinical parameters may offer a minimally invasive strategy for real‐time monitoring and personalized treatment in lung adenocarcinoma.

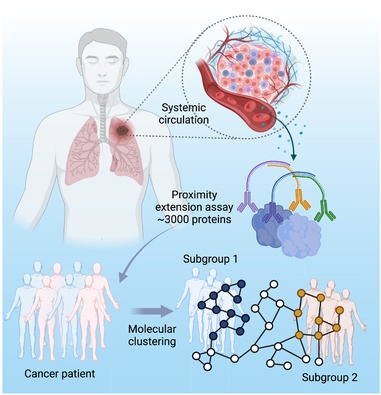

**Abbreviations**
AK1: Adenylate Kinase 1
BAX: Bcl‐2‐associated X protein
CASP1: Caspase 1
CASP8: Caspase 8
CASP9: Caspase 9
CCL20: Chemokine (C‐C motif) ligand 20
CEBPB: CCAAT/enhancer‐binding protein beta
CHAC2: Glutathione‐specific gamma‐glutamylcyclotransferase 2
CRKL: v‐Crk avian sarcoma virus CT10 oncogene homolog‐like
FOS: FBJ murine osteosarcoma viral oncogene homolog
GCLM: Glutamate‐cysteine ligase modifier subunit
GSEApy: Gene Set Enrichment Analysis in Python
IL‐17: Interleukin‐17
IQR: interquartile range
IRAK1: Interleukin‐1 Receptor‐Associated Kinase 1
iTRAQ: Isobaric Tags for Relative and Absolute Quantitation
LAP3: Leucine Aminopeptidase 3
JUN: Jun proto‐oncogene (Transcription factor, AP‐1 complex)
*k*
: *K* means algorithm
KEGG: Kyoto Encyclopedia of Genes and Genomes
LUAD: lung adenocarcinoma
MAP2K1: Mitogen‐Activated Protein Kinase Kinase 1/MEK1
MSigDB: Molecular Signatures Database
*n*
: sample size
NSCLC: non‐small cell lung cancer
*p*
: *p*‐value
PCA: principal component analysis
PEA: proximity extension assay
R: programming language
SMS: Spermine Synthase
STAT5B: Signal Transducer and Activator of Transcription 5B
TCGA: The Cancer Genome Atlas
TME: tumor microenvironment
TMT: Tandem Mass Tag
TNF: Tumor Necrosis Factor

## 1 INTRODUCTION

Lung adenocarcinoma (LUAD), the most common subtype of non‐small cell lung cancer (NSCLC), is associated with a poor 5‐year overall survival rate and considerable heterogeneity in genetic mutations, disease progression, and therapeutic response.^1^, ^2^ Although initial treatment often achieves short‐term success, relapse rates remain high.^3^ This heterogeneity is partly determined by the tumor microenvironment (TME), which plays a central role in cancer development, progression, and response to treatment.^4^ In particular, an immunosuppressive TME, often caused by chronic inflammation, has been associated with poor outcomes in lung cancer.^5^ While acute inflammation may promote anti‐tumor immune responses and cancer cell death, chronic inflammation contributes to immune evasion and supports tumor development, progression, and metastasis.^6^, ^7^ This complexity limits the effectiveness of uniform treatment strategies and underscores the need for personalized medicine.^1^

Beyond physiological, genomic, and transcriptomic data, high‐throughput omics technologies provide the ability to stratify patients based on molecular profiles. Cluster‐based stratification analysis of these datasets enables the identification of subgroups with common biological characteristics, improving the precision of personalized care.^8^ Specifically, systems‐level proteomics, refers to the large‐scale study of proteins in a biological system, including their abundance, and their interactions, to understand how they work together as an integrated network. Recent advances in systems‐level proteomics rely heavily on high‐resolution mass spectrometry in combination with data‐independent acquisition (DIA) approaches, multiplex labeling strategies (e.g., Tandem Mass Tag (TMT) and Isobaric Tags for Relative and Absolute Quantitation (iTRAQ)), and advanced bioinformatics for network reconstruction. Methods such as affinity proteomics (e.g., proximity extension assay [PEA]) enable the precise mapping of many proteins simultaneously in very small sample quantities and offer high potential for biomarker discovery.^9^ It provides a comprehensive overview of the disease phenotype and can reveal previously unrecognized subtypes. When applied to tumor or blood samples, this approach supports predictive analyses, early intervention, and evidence‐based decisions.^10^, ^11^

The Cancer Genome Atlas (TCGA) project laid the foundation for the molecular classification of NSCLC and identified subtypes for both lung squamous cell carcinoma and LUAD. More recently, proteomic and proteogenomic approaches have revealed new subtypes and potential therapeutic targets.^8^, ^9^, ^10^ However, translating these findings into clinical practice remains a challenge. Many studies focus on a single histologic subtype, which may exclude tumors with unclear or overlapping features, limiting generalizability. In addition, accurate prediction of postoperative outcomes remains difficult and requires the integration of multi‐omics data with robust clinical annotations. Most available multi‐omics datasets are from resected tumors. For patients with locally advanced NSCLC, adjuvant chemotherapy—usually platinum‐based doublets in combination with agents such as pemetrexed, gemcitabine, vinorelbine, or paclitaxel—remains the standard of care.^11^ Although this approach has improved survival rates, the molecular basis for treatment response remains poorly understood.^12^ Currently, there are no uniform biomarkers to predict the efficacy of adjuvant therapy, highlighting the need for deeper molecular characterization of clinically relevant patient cohorts. In this study, we subjected tumor samples from 88 LUAD patients to proteomic profiling and applied network‐based clustering to identify molecular subgroups. These clusters exhibited different immune signatures and recurrence patterns, giving us insights into the heterogeneity of LUAD and its clinical impact.

## 2 METHODS

### 2.1 Patient cohort

The plasma samples for the present study were collected from patients with adenocarcinoma of the lung, including 44 recurrent and 44 non‐recurrent samples. Cluster phenotypes were defined based on the demographic, clinical, and lifestyle data of the patients (see Table 1).

**TABLE 1:** Clinical and demographic descriptive statistics for the total population and two clusters.

Parameters	Overall cohort	Cluster 1	Cluster 2	*p* Value^a^
*n*	88	31	57	
Age in years, median (IQR)	64 (57–73)	67 (62–74)	61 (55–73)	.024
Sex, female, *n* (%)	58 (66%)	16 (52%)	42 (74%)	.037
Smoking status, *n* (%)				.002
Ex‐smoker	39 (44%)	14 (45%)	25 (44%)	
Current smoker	31 (35%)	5 (16%)	26 (46%)	
Non‐smoker	18 (21%)	12 (39%)	6 (10%)	
Smokers no of packet/year, median (IQR)^b^	37 (30–50)	40 (16–49)	37 (30–50)	.802
Recurrence, yes, *n* (%)	43 (49%)	12 (39%)	31 (54%)	.159
Recurrence month, median (IQR)^c^	37 (25–59)	56 (32–60)	31 (19–55)	.029
ECOG, *n* (%)	19	10	9	.073
Survival status, survived, *n* (%)	63 (72%)	22 (71%)	41 (72%)	.885
Survival in months, median (IQR)	48 (35–64)	53 (34–73)	47 (34–64)	.274
Comorbidities, *n* (%)				.389
COPD	16 (18%)	5 (16%)	11 (19%)	
Asthma	4 (4%)	1 (3%)	3 (5%)	
Emphysema	5 (6%)	2 (6%)	3 (5%)	
None	63 (72%)	23 (74%)	40 (70%)	

Abbreviations: COPD, Chronic obstructive pulmonary disease; ECOG, Eastern Cooperative Oncology Group; IQR, interquartile range.

^a^ *p*‐Values between the clusters.

^b^ Among the current smokers.

^c^ Among the recurrent patients, among the survived patients at the time of sampling.

### 2.2 Plasma secretome analysis

Plasma samples were prepared using the Olink® Explore 3 k protocol, which involves serial dilution of each sample to achieve the required input for one or more assay blocks of the Explore 3 k panels.^13^ We used the Olink® Explore platform, a high‐throughput multiplex immunoassay capable of quantifying approximately 3000 plasma proteins. This platform is based on PEA technology and was performed on the Illumina NovaSeq 6000 System (Illumina, USA). The detailed methodology was presented elsewhere.^13^

### 2.3 Data analysis

The proteomics dataset was generated in a single batch, and no technical batch effects were detected during quality assessment. Proteins with missing values across all samples (*n* = 44) were excluded from further analysis. This filtering step resulted in a final dataset containing 2894 proteins, of which 13 proteins had a missing value in only one sample; these proteins were retained in the analysis without imputation. To evaluate sample similarity, Euclidean distances were computed between all sample pairs using scaled proteomics data as input. The calculations were performed with the factoextra R package v1.0.7, and the resulting distance matrix was visualized as a heatmap to assess sample‐to‐sample relationships. *K*‐means clustering was performed using the implementation in the stats R package v4.4.0, as previously described.^14^ The optimal number of clusters (*k*) was determined using multiple validation metrics to ensure robust and reproducible results. The average silhouette width and Gap Statistic were calculated with factoextra v1.0.7, with higher values indicating better‐defined clusters. The Davies–Bouldin index^15^ was computed using the clusterCrit package v1.3.0, where lower values correspond to more compact and well‐separated clusters. The Calinski–Harabasz index was obtained via the fpc package v2.2.13, with higher scores indicating improved clustering performance. All metrics except the Davies–Bouldin index identified *k* = 2 as the optimal number of clusters, and this value was selected for subsequent analyses. To further validate clustering robustness, hierarchical clustering was performed using multiple linkage methods, including average, single, complete, Ward.D, and McQuitty. The hierarchical clustering results demonstrated high concordance with the *K*‐means solution, confirming the stability of the identified clusters. Finally, principal component analysis (PCA) was carried out using the PCAtools R package v2.16 to visualize the spatial distribution of samples in reduced dimensions, providing an orthogonal assessment of sample group separation.

### 2.4 Statistical analysis

A differential abundance analysis of proteins using the R package limma v3.60.2^16^ was performed after adjusting for age, sex, and smoking status to identify differentially regulated proteins between patient groups. Proteins with log2 fold change >1 or <−1 and adjusted *p*‐values <.001 were considered significantly regulated. The R package ComplexHeatmap v2.20.0^17^ was used to generate heatmaps to visualize the expression profile of the proteins. Gene Set Enrichment Analysis (GSEA) using the enrichr module from the Python package Gene Set Enrichment Analysis in Python (GSEApy) v1.1.3^18^, ^19^ was performed to find enriched pathways. The analysis was performed separately using the Kyoto Encyclopedia of Genes and Genomes (KEGG) pathways and the Molecular Signatures Database (MSigDB) Hallmark 2020 gene set as reference gene sets. To assess the robustness of the identified significant proteins, a permutation‐based significance testing approach was also applied. The class labels corresponding to the *K*‐means–defined clusters were randomly permuted 1000 times, and for each permuted dataset, differential expression analysis was performed using the limma modeling framework with identical covariate adjustments as in the original analysis. Significance was evaluated using predefined thresholds of adjusted *p*‐value <.001 (Benjamini–Hochberg correction) and |log_2_ fold change| >1. Across all permutations, no proteins met these criteria. Applying a less stringent threshold (adjusted *p*‐value <.05) similarly yielded no significant proteins in any permuted dataset. These results indicate that the significant proteins detected in the original dataset are unlikely to have arisen by chance. Effect size distributions were also compared between the real and permuted datasets without applying significance cutoffs. Density plots of log2 fold change values showed that the permuted datasets had logFC values centered near zero, with most values falling between −1 and 1. In contrast, the real dataset exhibited log2 fold change values concentrated between 1 and 2, with a higher peak density and no overlap with the permuted data distribution. This clear separation further supported the biological relevance of the observed differences.

## 3 RESULTS

### 3.1 Cluster identification based on plasma proteome profiles

**FIGURE 1:**
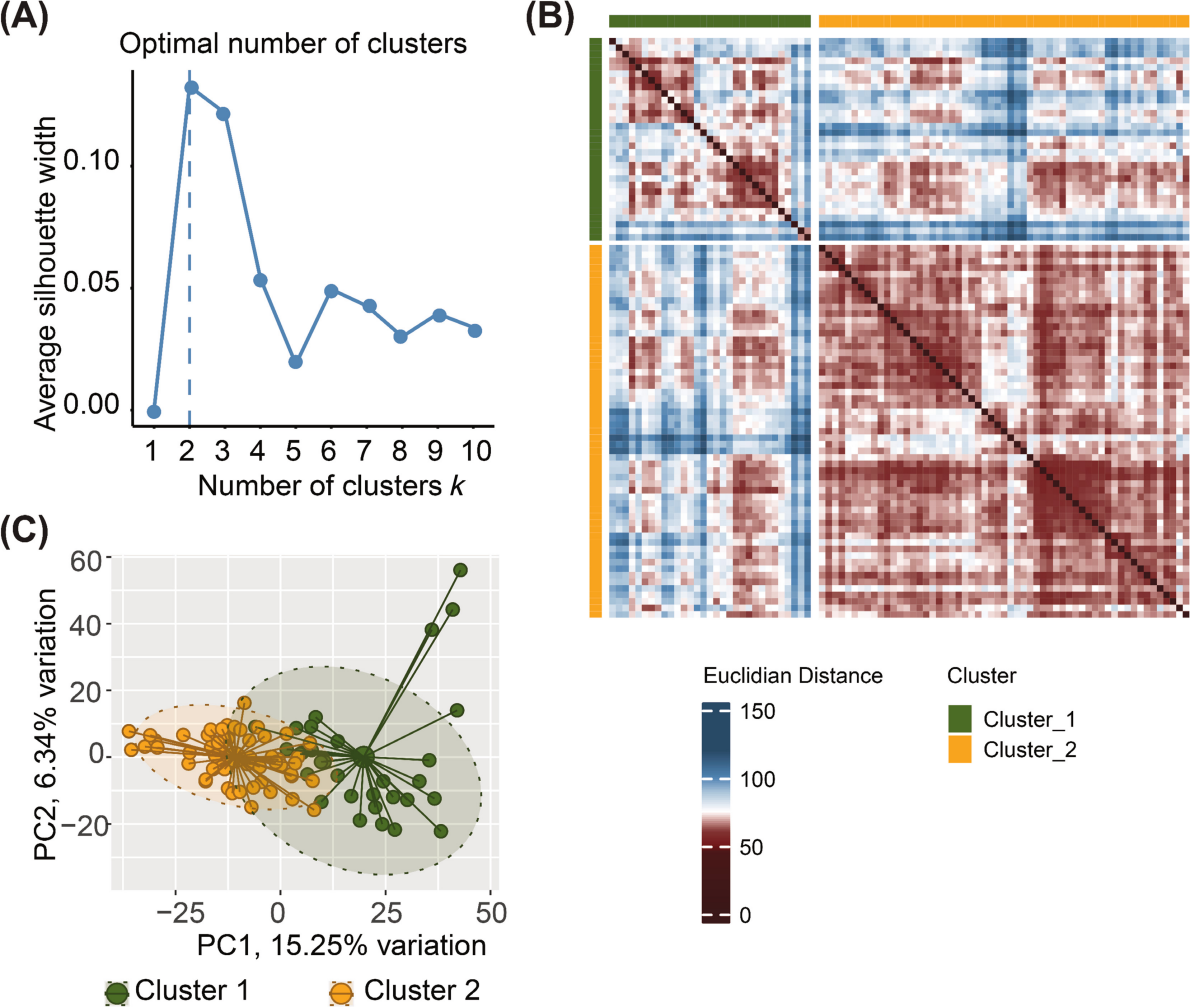
Data‐driven patient stratification and cluster identification and characterization. (A) Optimal number of patient clusters predicted based on average silhouette width. The prediction suggested the existence of two clusters of patients with maximum confidence. (B) Heatmap showing sample‐to‐sample similarities in the two predicted clusters. Rows and columns are samples, and the color scale represents the Euclidian distance between each pair of samples. The higher the distance, the lesser the similarity between samples. Top and left annotations show the predicted patient clusters associated with the samples. (C) Principal component analysis (PCA) plot visualizing the sample distribution. Samples are color‐coded for the predicted clusters (Green: Cluster 1 and orange: Cluster 2). Data ellipses denote 90% confidence space.

Unsupervised clustering using the silhouette method applied to 2894 proteins revealed that the optimal number of clusters was *k* = 2, as this gave the highest average silhouette width (Figure 1A). This analysis led to the identification of two clusters: Cluster 1 (*n* = 31) and Cluster 2 (*n* = 57) (Figure 1B). PCA, performed using the PCAtools package (v2.16.0), confirmed the separation of these two clusters (Figure 1C). The clinical and demographic characteristics of the clusters are summarized in Table 1. Statistically significant differences (*p* < .05) were observed for age, gender, and smoking status. Patients in Cluster 2 were younger (median [interquartile range, IQR]: 61 [55–73] vs. 67 [62–74] years, *p* = .024), had a higher proportion of women (74% vs. 52%, *p* = .037) and were more likely to be current smokers compared to Cluster 1 (46% vs. 16%, *p* = .002). Although the total number of recurrences did not differ significantly between the two clusters (*p* = .159), patients in Cluster 2 who experienced a recurrence had a significantly shorter median recurrence‐free survival (31 [19–55] vs. 56 [32–60] months, *p* = .029), suggesting a more aggressive clinical course. The complete list of differentially abundant proteins and pathway enrichment analysis by the KEGG and MSigDB Hallmark 2020 database can be found in Tables S1 and S2.

### 3.2 Differential protein expression and enrichment of immune pathways

**FIGURE 2:**
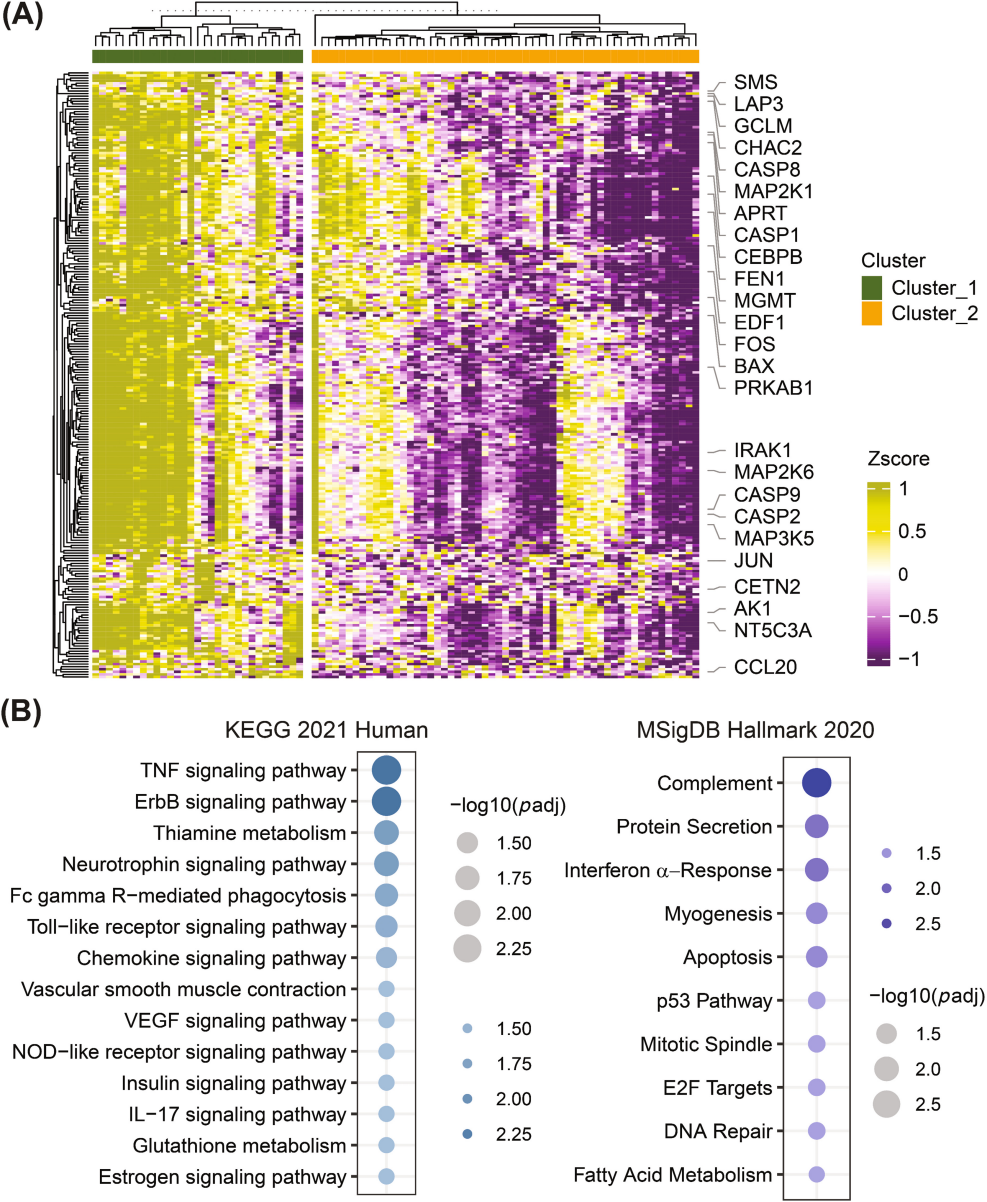
(A) Heatmap visualizing expression profile of 234 significantly abundant proteins (log fold change >1 and adj *p* < .001) in Cluster 1 compared to Cluster 2. Columns are samples, and rows are proteins. Column annotation represents the cluster, age, gender, and smoking status. (B) Bubble plot showing significantly enriched pathways in Cluster 1 compared to Cluster 2. The gene‐set enrichment analysis was performed by the Kyoto Encyclopedia of Genes and Genomes (KEGG) pathway and MSigDB Hallmark 2020 database.

To investigate the molecular basis of clinical differences between clusters, we performed differential protein abundance analysis using the limma package (v3.60.2), adjusted for age, sex, and smoking status. This identified 234 proteins whose abundance was significantly lower in Cluster 2 compared to Cluster 1 (log fold change >1, adjusted *p* < .001). A heatmap of these proteins was generated using ComplexHeatmap (v2.20.0) (Figure 2A). To interpret their biological relevance, we performed GSEA using the enrichr module in GSEApy (v1.1.3). Enrichment of KEGG pathways revealed significant involvement of immune and inflammatory pathways, including TNF and Toll‐like receptor signaling (e.g., Mitogen‐Activated Protein Kinase Kinase 1 / MEK1 (MAP2K1), Chemokine (C‐C motif) ligand 20 (CCL20), Caspase 8 (CASP8), Jun proto‐oncogene (Transcription factor, AP‐1 complex (JUN), FBJ murine osteosarcoma viral oncogene homolog (FOS), and Interleukin‐1 Receptor‐Associated Kinase 1 (IRAK1)), chemokine signaling (e.g., MAP2K1, CCL20, CRKL, and Signal Transducer and Activator of Transcription 5B (STAT5B)), glutathione metabolism (e.g. Spermine Synthase (SMS), Glutathione‐specific gamma‐glutamylcyclotransferase 2 (CHAC2), Glutamate–cysteine ligase modifier subunit (GCLM), and Leucine Aminopeptidase 3 (LAP3)) and Interleukin‐17 (IL‐17) signaling (e.g. CCL20, CASP8, JUN, FOS, and CCAAT/enhancer‐binding protein beta (CEBPB)) (Figure 2B). Analysis using the MSigDB Hallmark 2020 gene sets also showed enrichment in apoptosis (e.g., CASP8, Caspase 9 (CASP9), Caspase 1 (CASP1), and Bcl‐2‐associated X protein (BAX)) and the p53 signaling pathway (e.g., IRAK1, Adenylate Kinase 1 (AK1), BAX, FOS, and JUN). These results suggest that Cluster 2 has a largely suppressed immune and inflammatory profile, possibly due to a weakened immune response. This could impair anti‐tumor immunity and contribute to the shorter recurrence‐free survival observed in this group, indicating a more aggressive tumor phenotype.

## 4 DISCUSSION

This study demonstrates the potential of plasma proteomic profiling to reveal clinically significant subgroups in LUAD, the most common subtype of NSCLC. By integrating high‐throughput protein expression data with clinical and demographic characteristics, we identified two distinct molecular clusters among 88 LUAD patients. These subgroups differed significantly not only in their clinical characteristics, such as age, gender, and smoking status, but also in recurrence‐free survival, indicating important biological and prognostic differences.

While our analyses consistently supported a discrete two‐cluster (*k* = 2) solution, we recognize that the underlying biology may also follow a more continuous spectrum, with a gradient between two molecular extremes rather than fully distinct subgroups. Given the limited sample size and the nature of our dataset, we were not able to reliably apply supervised or archetypal approaches designed to capture such gradients. Future studies with larger cohorts and complementary data types will be essential to explore whether a continuous classification better reflects the underlying biology.

Our work builds on previous pioneering proteogenomic studies such as those by Soltis et al.^20^ and Gillette et al.,^10^ which integrated tumor‐based proteomics and genomics to identify LUAD subtypes and therapeutic vulnerabilities. While these studies used tissue‐based mass spectrometry and transcriptomics to characterize LUAD biology, our study demonstrates that meaningful stratification can also be achieved using plasma proteomics alone. The minimally invasive nature of blood sampling makes plasma proteomics an attractive option for repeated monitoring, in contrast to tissue biopsies. Ensuring reproducibility across different batches, instruments, and laboratories is an ongoing challenge that requires robust quality control measures and the use of internal standards. Efforts are currently underway to develop more cost‐effective and streamlined workflows for deep plasma proteome analysis, that could reduce the financial burden. The development of open peptide standards for plasma proteomics also aims to reduce costs and improve reproducibility.

This extends the utility of proteomics to a broader clinical setting, particularly when tissue availability is limited or long‐term follow‐up is required. In particular, we identified a cluster (Cluster 2) characterized by reduced immune and inflammatory signaling (e.g., TNF, Toll‐like receptor, and IL‐17 pathways) and enriched in younger, female and current smokers. This cluster also exhibited significantly shorter recurrence‐free survival, suggesting an aggressive tumor phenotype possibly related to a suppressed tumor immune microenvironment, a finding consistent with previous studies linking immunocold LUAD subtypes to poorer prognosis.^10^, ^20^, ^21^ In line with the recent work of Zhang et al.,^21^ who mapped the proteogenomic evolution of LUAD from preinvasive to invasive stages, our results emphasize the dynamic role of the immune system in disease progression. While Zhang et al. described four immune clusters in tissue‐based data, including immunodeficient phenotypes, we observed similar immunosuppression reflected in the circulating plasma proteome. These parallels support the idea that blood‐based proteomics can serve as a proxy for intratumoral immune states and could be used to guide treatment intensification or monitoring in high‐risk patients.

While our analyses consistently supported a discrete solution with two clusters, we recognize that the underlying biology may also follow a continuous spectrum, with a gradient between two molecular extremes and not completely distinct subgroups. Given the limited sample size and nature of our dataset, we were unable to reliably apply supervised or archetypal approaches to capture such gradients. Future studies with larger cohorts and complementary data types are essential to investigate whether continuous classification better reflects the underlying biology. This consideration is particularly important as plasma proteomics captures a systemic and potentially more dynamic picture of tumor–host interactions that may not correspond to strictly binary stratifications.

Our study is an important contribution to emerging efforts to refine LUAD classification beyond genomic mutations. Previous proteogenomic analyses have shown that alterations at the protein level are more strongly associated with clinical outcomes than mRNA alterations.^20^ Our data echo these conclusions and show that plasma protein signatures offer predictive insights into recurrence risk, potentially better than traditional clinical variables when adjusted for confounding factors such as age, sex, and smoking status.

The use of Olink's PEA‐based platform enabled the quantification of nearly 3000 proteins from small volumes of plasma, allowing for high throughput and reproducible measurements. Nevertheless, previous studies using the same platform in the context of immunotherapy have shown limited predictive value for treatment response.^22^ In contrast, our work focuses on prognosis and risk of recurrence rather than response to therapy and emphasizes the importance of systemic immunosuppression as an early biomarker of poor outcome in LUAD. Several limitations need to be considered. While the current sample size provides biological insight, larger and more diverse cohorts are needed to validate these findings and may limit the generalizability of the results. Although our study captures important signals from plasma, the lack of matched tumor tissue limits our ability to directly correlate systemic and local immune signatures. Future studies that integrate proteogenomics with multiple compartments, combining plasma, tumor, and tumor‐adjacent tissues—will be critical to build on this work.

In addition, while proteomic analysis identified potential pathways and protein markers, functional validation in experimental models is needed to confirm their role in tumor biology and response to therapy. These results lay the foundation for future prospective studies by identifying important molecular features that could serve as biomarkers or therapeutic targets. In addition, the knowledge gained could support the development of personalized therapeutic strategies by enabling stratification of patients based on proteomic profiles. Longitudinal data would also help to clarify the relationship between proteomic signatures and long‐term outcomes.

## 5 CONCLUSION

In summary, this study provides evidence that plasma‐based proteomic profiling can stratify LUAD patients into biologically and clinically distinct subgroups. These molecular phenotypes, particularly the immunosuppressed profile associated with poorer recurrence outcomes, offer insights into disease mechanisms and open new avenues for personalized treatment strategies in lung cancer.

## AUTHOR CONTRIBUTIONS

**Ujjwal Neogi:** Conceptualization; investigation; methodology; data curation; formal analysis; supervision; writing – original draft; writing – review and editing; visualization. **Anoop T. Ambikan:** Investigation; methodology; software; formal analysis; data curation; writing – review and editing; writing – original draft; visualization. **Kati Turkowski:** Methodology; writing – review and editing; writing – original draft. **Marc A. Schneider:** Methodology; data curation; writing – original draft; writing – review and editing. **Vanessa M. Beutgen:** Methodology. **Johannes Graumann:** Methodology; supervision; data curation; software; writing – original draft; writing – review and editing. **Hauke Winter:** Methodology; data curation; resources; writing – review and editing; writing – original draft. **Marek Bartkuhn:** Methodology; software; formal analysis; writing – review and editing; writing – original draft. **Werner Seeger:** Conceptualization; funding acquisition; supervision; writing – review and editing; writing – original draft. **Soni S. Pullamsetti:** Conceptualization; funding acquisition; supervision; resources; writing – review and editing; writing – original draft; investigation; project administration. **Rajkumar Savai:** Conceptualization; investigation; funding acquisition; writing – original draft; writing – review and editing; methodology; software; formal analysis; project administration; data curation; supervision; resources.

## FUNDING INFORMATION

This work was supported by Max Planck Society, Institute for Lung Health (ILH), the German Center for Lung Research (DZL, grant numbers 82DZL00402). SFB1213 (Project A01, A05 and A10N); the Excellence Cluster Cardio‐Pulmonary Institute (EXC 2026: Cardio Pulmonary Institute (CPI), project 390649896), State of Hesse (LOEWE iCANx, Project A6 B4, B5 and Area C), and European Research Council (ERC) Consolidator Grant (#866051). Ujjwal Neogi acknowledges support from the Swedish Research Council grant 2021‐01756 and Karolinska Institute Consolidator Grant (2‐117/2023).

## CONFLICT OF INTEREST STATEMENT

Ujjwal Neogi, Anoop T Ambikan, Kati Turkowski, Marc A. Schneider, Vanessa M. Beutgen, Johannes Graumann, Hauke Winter, Marek Bartkuhn, Soni S. Pullamsetti, and Rajkumar Savai have no conflicts of interest to declare. Werner Seeger has a potential personal conflict of interest as personal consulting fees from United Therapeutics, Tiakis, Lung Biotechnology PBC, Resyca BV, and Pfizer.

## ETHICS STATEMENT

The patients gave a written consent and the ethics committee of Giessen using samples from the lung biobank Heidelberg (study S‐270/2001) approved the study protocol for the investigation.

## Supporting information


**Table S1.** Differential protein abundance (LFC >1 and adj *p* < .001) between Clusters 1 and 2 after adjustment of age, sex, and smoking status.

**Table S2.** The gene‐set enrichment analysis using KEGG pathway and MSigDB Hallmark 2020 database with 234 significant proteins.

## Data Availability

The raw proteomics data generated using The Olink™ Explore is available in Figshare with https://doi.org/10.6084/m9.figshare.29064527.v1. R code is publicly available on GitHub (https://github.com/CollaborationSysVirol/AdenocarcinomaProteomics). Further information is available from the corresponding author upon request.
